# Effects of State-Level Earned Income Tax Credit Laws on Birth Outcomes by Race and Ethnicity

**DOI:** 10.1089/heq.2018.0061

**Published:** 2019-03-12

**Authors:** Kelli A. Komro, Sara Markowitz, Melvin D. Livingston, Alexander C. Wagenaar

**Affiliations:** ^1^ Department of Behavioral Sciences and Health Education, Rollins School of Public Health, Emory University, Atlanta, Georgia.; ^2^ Department of Epidemiology, Rollins School of Public Health, Emory University, Atlanta, Georgia.; ^3^ Department of Economics, Emory University, Atlanta, Georgia.

**Keywords:** birth outcomes, earned income tax credit, health disparities, health policy, infant health, socioeconomic factors

## Abstract

**Purpose:** Health disparities persist in birth outcomes by mother's income, education, and race in the United States. Disadvantaged mothers may experience benefit from supplements to family income, such as the earned income tax credit (EITC). We examined the effects of state-level EITCs on birth outcomes among women with a high school education or less, stratified by race and ethnicity.

**Methods:** A quasi-experimental multistate and multiyear difference-in-differences design is used to assess effects of the presence and generosity of 23 state-level EITC laws on birth outcomes from 1994 to 2013. The methods utilized the U.S. National Vital Statistics System birth data for the outcomes: birth weight, probability of low birth weight (LBW; <2500 g), and gestation weeks.

**Results:** Across all subgroups, any level of state EITC is associated with better birth outcomes with the largest effects seen among states with more generous EITCs. Black mothers experience larger percentage point reductions in the probability of LBW and increases in gestation duration. Among mothers with a high school education or less, results translate into 3760 fewer LBW babies with black mothers and 8364 fewer LBW babies with white mothers per year at the most generous state EITC level (i.e., 10% or more of federal and refundable). Hispanic and non-Hispanic mothers display relatively similar effects.

**Conclusions:** The EITC at the federal and state level is an effective policy tool to reduce poverty and improve birth outcomes across racial and ethnic subgroups. Given the historically higher risk among black mothers, state-level EITC expansions offer one policy option to address this persistent health disparity.

## Introduction

There are striking and persistent health inequities in birth outcomes by mother's income, education level, and race in the United States. Disparities by race are caused by a complex set of social factors across the life course, two of the most important being opportunities for quality education and employment. Compared with the majority white population, blacks are more than twice as likely to be poor (22% vs. 9%) and are less likely to have a college degree (23% vs. 36%).^1,2^ Such socioeconomic characteristics contribute to higher risks for negative birth outcomes among black women. Across racial/ethnic groups, a clear graded association exists between income quintile and low birth weight (LBW).^3^ This association persists with maternal education. Mothers with a high school education or less are at significantly higher risk for having a LBW infant compared to mothers with some college or more.^3^ In 2016, LBW levels ranged from 7% for births to non-Hispanic white women to nearly 14% among those to non-Hispanic black women.^4^ Rates among Hispanic subgroups, which are more similar to rates among whites than blacks, ranged from 7% for births to Mexican women to 9.5% for births to Puerto Rican women. Evidence suggests that inequities in birth outcomes between black and white mothers are caused by a lifetime of differential exposure to risks, including income inequality, educational achievement gaps, residential segregation, and toxic environmental exposures.^5,6^ The current study examines whether a key policy strategy to improve family income among low- to middle-income working families—the earned income tax credit (EITC)—improves birth outcomes similarly or differentially across race and ethnic subgroups.

The U.S. federal EITC has been credited as the largest and most effective antipoverty program for families in the United States.^7^ There is consistent evidence that federal and state EITCs positively affect families' economic circumstances; increase participation in the labor force, particularly among single mothers; reduce poverty, including child poverty; improve educational outcomes among children; and improve health outcomes among mothers and children.^8–10^ For example, multiple studies examining the effects of the federal^11,12^ and state^13–15^ expansions of the EITC on birth outcomes have found significant associations between the implementation of EITC laws with decreases in LBW and increases in average birth weight, especially among those of lowest socioeconomic status and the greatest increase in EITC income. We recently conducted a longitudinal study of changes in amount and refundability of EITCs across 23 states from 1994 to 2013 and found improvements in infant health outcomes (both birth weight and gestation weeks) in states with EITCs, with larger effects seen among states with more generous EITCs.^13^ We found little difference in maternal health behaviors (smoking and obtaining prenatal care) associated with state-level EITCs.

Given disparate birth outcomes by socioeconomic status and race, it is surprising that there is so little research on the effects of the EITC by race and ethnicity. In contrast to research documenting positive outcomes following federal and state EITC expansions,^8,9^ one study reported an iatrogenic effect of the federal EITC expansions during the 1990s on infants born in California to non-Hispanic black women who were eligible for Medicaid and had a high school education or less.^16^ Specifically, 2 months after EITC disbursement, there was an increase in the odds of very LBW (<1500 g) among infants born to black women.^16^ However, this association was not significant at 3 or 4 months after EITC disbursement. A similar pattern was observed among births to non-Hispanic white women in California, although the effect was smaller. It is difficult to understand why the results of this study are inconsistent with the majority of the extant literature, which finds beneficial effects of EITCs.

A more comprehensive study of the effects of federal expansions of the EITC during the 1990s examined effects across all states on similar “high impact” mothers (single women with high school education or less). The study results indicated that the federal EITC expansions reduced the likelihood of having a LBW baby (<2500 g) for black mothers by 0.73 percentage points (relative to a mean of 14.4%); this is more than four times larger than the effect on white mothers (0.13 percentage point decline relative to a mean of 8.1%).^7,12^ EITC effects were smaller among Hispanic mothers than non-Hispanic mothers, perhaps because Hispanic children tend to have better baseline birth outcomes and some do not qualify for the EITC as undocumented immigrants. They suggest that the differences by race may be largely attributed to the fact that black mothers are more at risk, having almost double the risk of low-birth weight births and lower income. Results of their study imply that the EITC may be a policy strategy that will not only contribute to reducing negative birth outcomes but also birth inequities.

We previously evaluated effects of the presence and generosity of 23 state-level EITC laws on maternal health behaviors and birth outcomes from 1994 to 2013 among women with a high school education or less. We found few statistically significant differences in maternal health behaviors associated with state-level EITC. By contrast, results for infant health outcomes of birth weight and gestation weeks consistently showed improvements in states with any level of EITC and larger effects within states with more generous EITCs.^13^ The current study expands upon our previous study by examining differential effectiveness of the EITC across race and ethnic subgroups. Black mothers are at greatest risk of low income, low educational attainment, and poor birth outcomes, and, therefore, may experience greater benefit from supplements to family income. Given the increased risk among black mothers and their babies, and the larger federal EITC effect among blacks found by Hoynes et al.,^12^ we hypothesize that the beneficial effect of a state EITC will be larger among black babies.

## Methods

### Earned income tax credit

To ensure reliable and valid indicators of state policy changes from 1994 to 2013, we conducted original legal research on EITC policy for each of the 50 states, plus Washington DC. In collaboration with a team of legal researchers, we developed a codebook and detailed coding protocol to capture important EITC policy dimensions, including eligibility criteria and amount and refundability of the credit. Quality control procedures included blinded independent coding of a random sample of items by two trained legal researchers. All coders were closely supervised by a senior attorney, who reviewed protocols with coders for any variable showing 5% or higher cross-coder disagreement rate. All divergences between two coders were resolved by the supervising attorney after meeting with the two coders and examining the original legal text.

If the state has an EITC, the value is usually expressed in the law as a percent of the federal EITC, but some states specify a dollar amount of the credit which we converted to a percent of federal based on the relevant federal dollar amount. The values of the state credits often vary based on number of children living in the household. In addition, some states specify that the EITC is refundable, meaning that if tax liability falls to zero, the government will send a refund check for the credit amount. Nonrefundable credits provide no further income beyond a zero tax liability.

We use the information gathered to create a series of indicators combining the presence of and generosity of EITC payments as follows: (1) states with no EITC (reference category); (2) states with an EITC, nonrefundable payments, and payments less than 10% of the federal amount; (3) states with an EITC, payments that are refundable, and payments less than 10% of the federal amount; (4) states with an EITC, nonrefundable payments, and payments 10% or more of the federal amount; and (5) states with an EITC, refundable payments, and payments 10% or more of the federal amount.^13^ We use the 10% cutoff because this is the median value of EITC among states over the sample period. In 1994, 5 states had an EITC in place, but by 2013 this number grew to 26 states, plus Washington DC. Maryland is excluded from analysis because of the unique structure of their EITC law, which does not match the measurement model used for all other states. Our legal research identified 80 changes in state EITC law from 1994 to 2013. After condensing into our 5 policy categories, the 80 legal changes represented 34 shifts in policy category across 23 states over the study period. Variable operationalization details have been previously reported.^13^ Washington state enacted a refundable credit of 5% of the federal EITC in tax year 2009 and was scheduled to rise to 10% in 2010. Due to state budget shortfalls, policy makers have not yet financed the credit. Therefore, we placed Washington state into the no EITC policy category.

### Birth outcomes

Infant health outcomes and maternal characteristics come from the U.S. National Vital Statistics System, which records a 100% census of U.S. births annually. Infant health outcomes include birth weight, probability of LBW (less than 2500 g), and gestation weeks. We cannot identify the individual women who assuredly qualify for the EITC, instead we use mother's education to limit the sample and estimate models that represent an approximation of intent-to-treat.^13^ We also limit the sample to singleton births, and we do not include young teenage mothers (women less than age 18).

### Covariates

Maternal characteristics recorded on birth certificates include: mother's age, marital status, mother's education, and mother's race/ethnicity. In addition, we use county geographic identifiers to merge county-level covariates potentially related to the outcomes. These include the county unemployment rate, real income per capita, percent poverty, number of obstetricians/gynecologists and primary care physicians per 1000 women ages 15–44, and county population size. Specific county characteristics are unavailable for counties of less than 100,000 people. For these small counties, we use the average value for all counties in the state with a population less than 100,000. All models also include state fixed effects and year-by-quarter fixed effects. Summary statistics by race (black, white) and ethnicity (Hispanic, non-Hispanic) for all variables included in the models are shown in Table 1.

**Table 1.** Means and Standard Deviations of Study Variables by Race and Ethnicity

Variables	Black	White	Hispanic	Non-Hispanic
Dependent variables: infant health				
Birth weight	3104 (621)	3321 (560)	3322 (546)	3260 (593)
Birth weight <2500 g	0.12 (0.32)	0.06 (0.24)	0.06 (0.23)	0.08 (0.27)
Gestation weeks	38.30 (3.06)	38.85 (2.45)	38.80 (2.45)	38.72 (2.65)
State EITC variables (no state EITC omitted reference)				
Low EITC no refund	0.03 (0.18)	0.04 (0.20)	0.04 (0.19)	0.04 (0.20)
Low EITC with refund	0.05 (0.21)	0.05 (0.22)	0.04 (0.19)	0.06 (0.24)
High EITC no refund	0.01 (0.10)	0.01 (0.07)	0.004 (0.06)	0.01 (0.08)
High EITC with refund	0.15 (0.36)	0.11 (0.32)	0.11 (0.31)	0.13 (0.33)
Individual-level covariates				
Maternal age	24.73 (5.63)	25.77 (5.70)	26.13 (5.77)	25.28 (5.64)
Married	0.21 (0.41)	0.55 (0.50)	0.50 (0.50)	0.48 (0.50)
Female baby	0.49 (0.50)	0.49 (0.50)	0.49 (0.50)	0.49 (0.50)
Black	1.00 (—)	0.00 (—)	0.03 (0.17)	0.27 (0.44)
Hispanic	0.05 (0.23)	0.41 (0.49)	1.00 (—)	0.00 (—)
Less than high school	0.34 (0.47)	0.39 (0.49)	0.58 (0.49)	0.27 (0.45)
County-level covariates				
Unemployment	6.29 (2.46)	6.20 (2.80)	6.69 (3.21)	5.96 (2.42)
Real income per capita (in $1000s)	17.12 (5.00)	16.29 (4.48)	17.38 (5.17)	15.93 (4.15)
Percent poverty	15.99 (5.27)	14.26 (5.37)	15.59 (6.00)	14.04 (4.96)
Primary care physicians per 1000 females age 15–44	1.97 (0.74)	1.88 (0.69)	1.84 (0.60)	1.93 (0.75)
County pop 500,000–1,000,000	0.22 (0.42)	0.15 (0.36)	0.18 (0.39)	0.16 (0.36)
County pop 250,000–500,000	0.15 (0.35)	0.14 (0.35)	0.13 (0.34)	0.15 (0.35)
County pop 100,000–250,000	0.13 (0.34)	0.16 (0.36)	0.10 (0.30)	0.18 (0.38)
County pop <100,000	0.18 (0.38)	0.28 (0.45)	0.11 (0.32)	0.34 (0.47)
No. of observations	5,371,607	23,898,390	10,176,407	18,914,603

EITC, earned income tax credit.

### Statistical methods

Birth certificate data and state EITC data were merged based on the number of prior children and outcome specific theory driven lags. For maternal outcomes, we merged EITCs from the year before conception. For infant outcomes, we merged the EITC received closest to the time of birth.

We estimated race and ethnicity specific effects of state EITCs using fully-interacted, fixed-effect panel data models. That is, all covariates described above enter the model after being multiplied by the individual-specific indicator variables for race (black and white) and, subsequently, by ethnicity (Hispanic and non-Hispanic). All models were estimated as linear probability models for ease of interpretation of the estimates. Models were re-estimated as logistic regressions without any substantive changes. All models accounted for within-state serial correlation by clustering the standard errors at the state level.

The equality of the race and ethnic specific estimates for each of the EITC indicators was tested with a F-test of the interacted coefficients. We respecified the model to generate coefficients that reflect the additional impact of EITC for blacks (Hispanics) differing from the white (non-Hispanic) effect and conducted an omnibus F-test of this set of black (Hispanic) EITC coefficients. The results of these tests are reported in the text below and in the notes to the table.

These fixed effects isolate the effect of state EITCs so that our EITC indicator estimates reflect the average difference before and after the change in each policy within states, after subtracting the before and after differences in states without a state-specific EITC, effectively treating states without a state-specific EITC as a control group. To bias our estimates of the effect of state-specific EITCs, potential confounders would need to change differentially over time between states, and these changes would need to occur at a similar time to changes in our state-specific EITC indicators. To protect against such confounding, we included the previously discussed individual-level and county-level covariates in all models. Birth certificate data does not allow us to ascertain which individual women qualify for the EITC. To better approximate and intent-to-treat analysis, we restrict all models to women who have reported a high school education or less.

## Results

Table 2 shows results by mother's race, controlling for Hispanic ethnicity, for the effect of state-level EITCs on birth outcomes among women with a high school education or less. Across race and consistent with our previous report,^13^ any level of state EITC is associated with improved birth outcomes, and the largest effects are seen among states with more generous EITCs (Table 2).

**Table 2.** Effects of Presence and Generosity of Earned Income Tax Credit Laws, Low Educated Black Versus White Mothers

	Birth weight		*p*-Value black vs. white mothers	Birth weight <2500 g		*p*-Value black vs. white mothers	Gestation weeks		*p*-Value black vs. white mothers
	Black	White		Black	White		Black	White	
Low EITC no refund	16.120^***^ (5.926)	9.380^***^ (3.446)	0.1730	−0.007^***^ (0.001)	−0.002^**^ (0.001)	0.0005	0.115^***^ (0.025)	0.035 (0.027)	0.0000
Low EITC with refund	19.342^***^ (6.830)	18.219^**^ (7.094)	0.8410	−0.009^***^ (0.003)	−0.005^***^ (0.002)	0.1063	0.086 (0.056)	0.016 (0.032)	0.0274
High EITC no refund	19.572^***^ (5.132)	10.462^***^ (3.894)	0.0216	−0.006^**^ (0.003)	−0.002^***^ (0.001)	0.0433	0.176^***^ (0.016)	0.161^***^ (0.017)	0.3000
High EITC with refund	37.164^***^ (7.967)	28.400^***^ (6.923)	0.2912	−0.014^***^ (0.003)	−0.007^***^ (0.002)	0.0181	0.148^**^ (0.056)	0.066^**^ (0.031)	0.0730
Overall *N*	29,269,997			29,269,997			29,290,045		
Race-specific *N*	5,371,607	23,898,390		5,371,607	23,898,390		5,377,596	23,912,449	

Robust, state-clustered standard errors in parentheses. ^*^p<0.10, ^**^p<0.05, ^***^p<0.01.

*p*-Values for the omnibus F-test of the statistical significance of the set of EITC coefficients for black differing from the white coefficients are 0.1839 for birth weight; 0.0048 for birth weight <2500 g; and 0.0001 for gestation.

Birth weight gains are higher for black mothers in states with an EITC (16.12–37.16 g representing 0.5–1.2% increases in birth weight) than for white mothers (9.38–28.40 g representing 0.3–0.9% increases in birth weight), but were not significantly different between black compared with white mothers, except among states with a high EITC with no refund.

Results show a larger beneficial effect among black mothers compared with white mothers for the probability of LBW (omnibus test for set of 4 EITC×black interactions *p*=0.0048) and gestation weeks (omnibus test *p*=0.0001). Reductions in the probability of LBW for black mothers in states with an EITC range from 0.6 to 1.4 percentage points (depending on size and refundability of the credit), which represent 5–11.7% reductions from the mean of 12%. Reductions in probability of LBW for white mothers in states with an EITC range from 0.2 to 0.7 percentage points, which represent 3.3–11.7% reductions in the mean of 6%. Among black mothers with a high school education or less, these results translate into 1611–3760 fewer babies born LBW to the 268,580, on average, singleton and nonteenage births per year in the United States. Among white mothers with a high school education or less, these results translate into 2390–8364 fewer babies born LBW to the 1,194,920, on average, singleton and nonteenage births per year.

Average gestation weeks improve for black and white mothers with high-value state EITCs (i.e., 10% or more of federal with or without a refund). Among black mothers in states with high-value EITCs, on average, gestation weeks increase by small fractions of a week, representing 0.38–0.46% longer gestation weeks. The comparable numbers among white mothers in states with high-value EITCs are 0.17–0.41% longer gestation weeks.

Table 3 shows results by mother's Hispanic ethnicity, controlling for race, for the effect of state-level EITCs on birth outcomes among women with a high school education or less. There are few statistically significant differences in effects by ethnicity, with the exception of one category of EITC for LBW and another for gestation weeks. Overall, increases in birth weights for Hispanic mothers in states with an EITC range from 11 to 36 g representing 0.3–1.1% increases in birth weight, which is similar for non-Hispanic mothers with a range of 9–28 g representing 0.3–0.9% increases in birth weight. Reductions in the probability of LBW among babies with Hispanic mothers in states with an EITC range from 0.1 to 0.7 percentage points, which represent 1.7–11.7% reductions in the mean of 6%. Similarly, reductions in the probability of LBW among babies with non-Hispanic mothers in states with an EITC range from 0.4 to 0.9 percentage points, which represent 5–11.3% reductions in the mean of 8%. Among Hispanic mothers with a high school education or less, these results translate into 508–3562 fewer babies born LBW to the 508,820, on average, singleton and nonteenage births per year in the United States. Among non-Hispanic mothers with a high school education or less, these results translate into 3782–8512 fewer babies born LBW to the 945,730, on average, singleton and nonteenage births per year.

**Table 3.** Effects of Presence and Generosity of Earned Income Tax Credit Laws, Low Educated Hispanic Versus Non-Hispanic Mothers

	Birth weight		*p*-Value Hispanic vs. non-Hispanic mothers	Birth weight <2500 g		*p*-Value Hispanic vs. non-Hispanic mothers	Gestation weeks		*p*-Value Hispanic vs. non-Hispanic mothers
	Hispanic	non-Hispanic		Hispanic	non-Hispanic		Hispanic	non-Hispanic	
Low EITC no refund	11.264^***^ (3.687)	8.667^**^ (4.100)	0.5353	−0.001^*^ (0.001)	−0.004^***^ (0.001)	0.0227	0.051^*^ (0.030)	0.045 (0.027)	0.6961
Low EITC with refund	21.431^***^ (6.502)	15.530^*^ (8.229)	0.3621	−0.004^**^ (0.002)	−0.006^**^ (0.002)	0.1167	0.055 (0.037)	0.007 (0.037)	0.0582
High EITC no refund	16.561^***^ (5.716)	11.851^***^ (4.144)	0.3158	−0.002^**^ (0.001)	−0.003^**^ (0.001)	0.5640	0.086^***^ (0.014)	0.190^***^ (0.014)	0.0000
High EITC with refund	35.613^***^ (7.306)	28.492^***^ (7.675)	0.3704	−0.007^***^ (0.002)	−0.009^***^ (0.002)	0.4979	0.112^**^ (0.045)	0.068^**^ (0.030)	0.2307
Overall *N*	29,091,010			29,091,010			29,110,719		
Race-specific *N*	10,176,407	18,914,603		10,176,407	18,914,603		10,180,789	18,929,930	

Robust, state-clustered standard errors in parentheses, ^*^p<0.10, ^**^p<0.05, ^***^p<0.01. *p*-Values for the omnibus F-test of the statistical significance of the set of EITC coefficients for Hispanic differing from the non-Hispanic coefficients are 0.8367 for birth weight; 0.2141 for birth weight <2500 g; and 0.0000 for gestation.

Finally, average gestation week increases are also similar for Hispanic and non-Hispanic mothers, with a high-value refundable state EITC increasing average gestation weeks by small fractions of a week, representing 0.2–0.3% increases. In states with a high-value nonrefundable EITC, Hispanic mothers have a lower gestation week increase (0.6 day, 0.22% increase) compared with non-Hispanic mothers (1.33 days, 0.49% increase).

## Discussion

Consistent with previous research,^7,12^ including our own study of state EITCs,^13^ we find beneficial effects of the EITC on birth outcomes across race and ethnic subgroups. We find larger percentage-point effects for black mothers with a high school education or less; however, given their higher baseline rate, the relative percent reduction is similar among black and white mothers. In states with the most generous state EITCs, refundable and 10% or more of the federal, we find nearly 12% reductions in LBW births for black and white mothers. Among mothers with a high school education or less, this reduction translates to 3760 fewer babies born LBW with black mothers and 8364 fewer babies with white mothers per year across the United States. In addition, gestation time is slightly longer and birth weights are higher among babies with black mothers. We find few differences in effects by ethnicity, including an inconsistent pattern of effects on gestation time.

The direction and magnitude of these results by race are similar to those found for the federal expansions of the EITC, with larger absolute beneficial effects for black mothers given their higher baseline rate of risk.^7,12^ The federal EITC expansions resulted in smaller treatment effects among Hispanic than non-Hispanic mothers. Hoynes et al. attribute this finding to a lower baseline rate of low birth rate among Hispanic mothers, speculating that there was less room for improvement.^12^ In our study, we find few statistically significant differences by ethnicity, although patterns are consistent with the findings of the federal EITC.

We use a strong quasi-experimental design to study the effects of size and type of state-level EITCs on birth outcomes by race and ethnicity. This study design controls for time trends and state differences in birth outcomes, as well as other important covariates. A limitation is the inability to specifically identify individual women who assuredly qualify for the EITC—instead we use mother's education to limit the sample and estimate models that represent an approximation of intent-to-treat. Another limitation is that the observed results on LBW could be an underestimation of the differential effect, given that black women have a rate of stillbirth twice higher than white women, and stillbirths are excluded from our data on live births.^17^ In summary, our results are consistent with previous research finding overall beneficial effects of the EITC on birth outcomes across race and ethnicity.

## Conclusion

The federal and state EITC is an effective policy tool to reduce poverty and improve birth outcomes across racial and ethnic subgroups. Given ongoing disparities, it remains essential to advance policy options that may help reduce these disparities and explore other policy options that may prevent and further mitigate the effects of social disadvantage across the life course for those mothers most at risk.

## Abbreviations Used

EITC: earned income tax credit
LBW: low birth weight
